# Inequality as a Powerful Predictor of Infant and Maternal Mortality around the World

**DOI:** 10.1371/journal.pone.0140796

**Published:** 2015-10-21

**Authors:** Juan Ignacio Ruiz, Kaamel Nuhu, Justin Tyler McDaniel, Federico Popoff, Ariel Izcovich, Juan Martin Criniti

**Affiliations:** 1 Department of Health Education and Recreation, College of Education and Human Services, Southern Illinois University Carbondale, Carbondale, Illinois, United States of America; 2 Servicio de Clínica Médica, Hospital Aleman, Ciudad Autónoma de Buenos Aires, Argentina; Institute for Health & the Environment, UNITED STATES

## Abstract

**Background:**

Maternal and infant mortality are highly devastating, yet, in many cases, preventable events for a community. The human development of a country is a strong predictor of maternal and infant mortality, reflecting the importance of socioeconomic factors in determinants of health. Previous research has shown that the Human Development Index (HDI) predicts infant mortality rate (IMR) and the maternal mortality ratio (MMR). Inequality has also been shown to be associated with worse health in certain populations. The main purpose of the present study was to determine the correlation and predictive power of the Inequality Adjusted Human Development Index (IHDI) as a measure of inequality with the Infant Mortality Rate (IMR), Maternal Mortality Rate (MMR), Early Neonatal Mortality Rate (ENMR), Late Neonatal Mortality Rate (LNMR), and the Post Neonatal Mortality Rate (PNMR).

**Methods and Findings:**

Data for the present study were downloaded from two sources: infant and maternal mortality data were downloaded from the Global Burden of Disease 2013 Cause of Death Database and the Human Development Index (HDI) and Inequality-Adjusted Human Development Index (IHDI) data were downloaded from the United Nations Development Program (UNDP). Pearson correlation coefficients were estimated, following logarithmic transformations to the data, to examine the relationship between HDI and IHDI with MMR, IMR, ENMR, LNMR, and PNMR. Steiger’s Z test for the equality of two dependent correlations was utilized in order to determine whether the HDI or IHDI was more strongly associated with the outcome variables. Lastly, we constructed OLS regression models in order to determine the predictive power of the HDI and IHDI in terms of the MMR, IMR, ENMR, LNMR, and PNMR.

Maternal and infant mortality were both strongly and negatively correlated with both HDI and IHDI; however, Steiger’s Z test for the equality of two dependent correlations revealed that IHDI was more strongly correlated than HDI with MMR (*Z* = 4.897, *p* < 0.001), IMR (*Z* = 2.524, *p* = 0.012), ENMR (*Z* = 2.936, *p* = 0.003), LNMR (*Z* = 2.272, *p* = 0.023), and PNMR (*Z* = 2.277, *p* = 0.023). Furthermore, side-by-side OLS regression models revealed that, when IHDI was used as the predictor variable instead of HDI, the *R* ^*2*^ value was 0.053 higher for MMR, 0.025 higher for IMR, 0.038 higher for ENMR, 0.029 higher for LNMR, and 0.026 higher for PNMR.

**Conclusions:**

Even when both the HDI and the IHDI correlate with the infant and maternal mortality rates, the IHDI is a better predictor for these two health indicators. Therefore, these results add more evidence that inequality is playing an important role in determining the health status of various populations in the world and more efforts should be put into programs to fight inequality.

## Introduction

Maternal and child mortality are devastating events for any community, which is why children and expectant mothers are priority populations for many public health intervention programs the world over. Maternal mortality is unacceptably high, with an estimated 800 women dying from pregnancy or childbirth-related complications around the world every day and almost all maternal deaths (99%) occur in developing countries, of which more than half occur in sub-Saharan Africa [1]. Sadly, the overwhelming majority of these deaths are needless and readily preventable. With regard to childhood mortality, an estimated 6.3 million children under the age of 5 died in 2013 alone, and like maternal mortality, most of these deaths were preventable [2].

Maternal and childhood mortality remain two of the most important health status indicators of any community. They reflect and are affected by the nutritional status of women and children, access to and quality of healthcare, immunization status, conditions of housing, income, and level of education [3–5]. Therefore, the degree of development of a population will predict the infant and maternal mortality of that population. In 1997 Lee et al. showed that the Human Development Index (HDI) could serve as a powerful predictor of both infant and maternal mortality [6].

The HDI is a statistical tool used to assess the degree of well-being and quality of life of a population [7]. Participating countries are ranked according to their level of human development relative to others. This index takes into account three indicators: education, health, and income. The education component of the index is represented by the adult literacy rate. The health component of the index is represented by life expectancy at birth (LEB) and the per capita gross domestic product (GDP) represents the income. These indicators—such as GDP—are national aggregates, and, thus, may not exactly reflect their distribution in the general population. By extension, therefore, the HDI does not sufficiently depict the distributional inequalities of health, education, and income within these countries. For this reason, in 1997, Hiscks developed a new index incorporating the inequality factor, calling it the Inequality adjusted Human Development Index (IHDI) [8]. According to the United Nations Development Program (UNDP), the IHDI takes into account how education, health, and income achievements are distributed among the population of each country and the difference between the IHDI and HDI informs the loss of human development due to inequality [9].

To the extent that earlier works showed evidence supporting the association between worse health outcomes and higher inequality among populations [10–15], this paper seeks to explore and compare the strengths of association between the HDI and IHDI. Specifically, the objectives of the present article are: 1) to determine the correlation between the HDI and both the infant and maternal mortality rates; 2) to determine the correlation between the IHDI and both the infant and maternal mortality rates; and 3) to compare the predictive power of the HDI with the IHDI for both infant and maternal mortality rate.

## Methods

Data for the present study was obtained from two sources. Firstly, data on maternal and infant (early neonatal, late neonatal, and post neonatal) mortality rates for 188 countries for the year 2013 were obtained from the 2014 Global Burden of Disease 2013 Cause of Death Database [16,17]. Data for the Human Development Index (HDI) and Inequality-Adjusted Human Development Index (IHDI) were obtained for the year 2013 from the United Nations Development Program (UNDP) website [18]. After initial assessment of the data from the UNDP website, it was realized that IHDI data for some countries was missing. Subsequently, the sample sizes were adjusted, culminating in 145 final observations (*n* = 145).

Before describing the analytical methods employed for the present study, it may be useful to offer some definitions of the dependent variables. Regarding infant mortality, the “under-five mortality rate” which was the variable utilized in the present study as a measure of the infant mortality rate (IMR)–was defined as the number of children who died by the age of five for every 1,000 live births per year. In other words, the “under-five mortality rate” represented the probability per 1,000 that a newborn baby would die before reaching the age of five years. The early neonatal mortality rate (ENMR) measures the probability of infant mortality within seven days of birth, the late neonatal mortality rate (LNMR) measures probability of infant mortality within seven to 28 days following birth and the post-neonatal mortality rate (PNMR) measures the probability of infant mortality within 28 days to one year following birth.

We used data from 145 countries (*n* = 145) to calculate Pearson correlation coefficients and OLS regression models between the HDI and MMR, IMR, ENMR, LNMR, and PNMR. We also used data from 145 countries (*n* = 145) in order to calculate Pearson correlation coefficients and OLS regression models between IHDI and MMR, IMR, ENMR, LNMR, and PNMR S1 Table. In order to determine whether HDI or IDHI correlated more strongly with various mortality measures, correlation coefficients between (a) HDI and all mortality indicators and (b) IHDI and all mortality indicators were tested for differences by utilizing Steiger’s Z test for equality of two dependent correlations [19].

To the extent that the infant and maternal mortality variables were not normally distributed according to Kolmogorov-Smirnov test for normality, transformations were applied to the data in an attempt to achieve normality. A natural log transformation was applied to the MMR, IMR, ENMR, LNMR, and the PNMR variables. Therefore, the regression equation used to model the aforementioned variables took the following form:

$$Ln(Y_{1})=\;\alpha +\;\beta_{1}X_{1}+\;\varepsilon_{1}$$

Further testing with the Kolmogorov-Smirnov test revealed that, even after data transformation, the data failed to reach normality. While analysis of histogram outputs revealed that each variable approached normality following transformation, we elected to take supplementary precautions in handling the data. Specifically, correlation coefficients and beta coefficients were estimated with bootstrapped (1,000 resamples) 95 percent bias corrected confidence intervals. We also downloaded polygon feature data for a map of the world [20] and utilized ESRI ArcGIS version 10.0 [21] to project maps with the following layers: MMR, IMR, HDI, and IHDI.

## Results

### Descriptive Data

**Fig 1:**
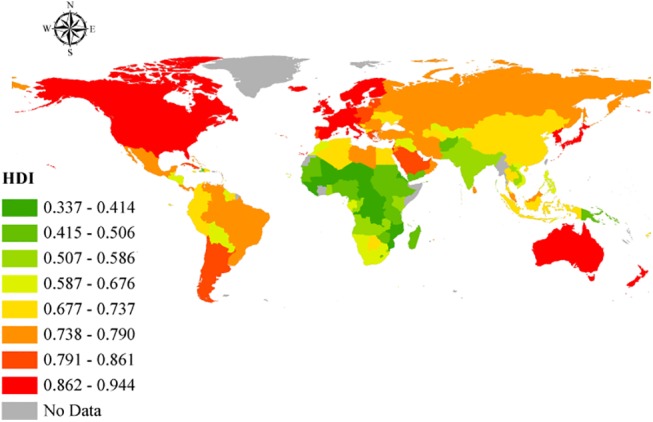
Geographic representation of the HDI. Open source polygon feature data for the maps were downloaded [20] and, subsequently, projected using ESRI ArcGIS [21].

**Fig 2:**
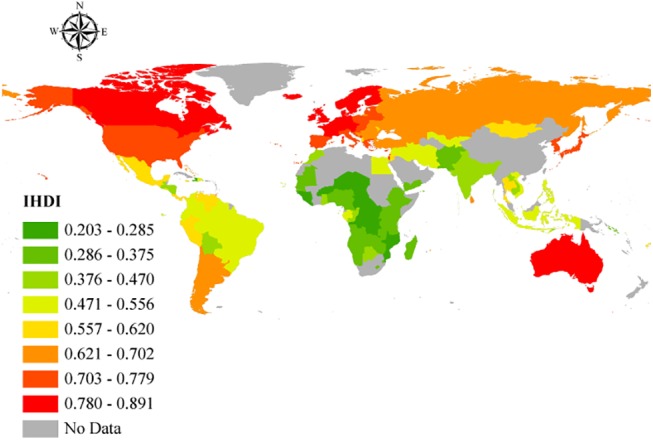
Geographic representation of the IHDI. Open source polygon feature data for the maps were downloaded [20] and, subsequently, projected using ESRI ArcGIS [21].

With regard to human development, significant range in HDI can be observed internationally (Fig 1). When adjusted for inequalities (Fig 2), there were changes in the position that some of the countries occupied in the HDI rank–as specified by the United Nations Development Programme (i.e., low, medium, high, and very high) [18]. For the 49 countries with very high human development, there were 17 countries that differed in ranks after adjusting for inequality. Four countries from the aforementioned group had a percentage difference between the IDHI and the HDI higher than 15% (i.e., Chile, Republic of Korea, United States of America, and Argentina, 19.6 percent, 17.4 percent, 17.4 percent, and 15.3 percent, respectively). The average percentage of overall loss was 10.4 percent for the countries with very high human development. With regard to the 43 countries in the group with low human development, the percentage difference between the IHDI and the HDI was in the range of 23.8 percent and 44.3 percent.

**Fig 3:**
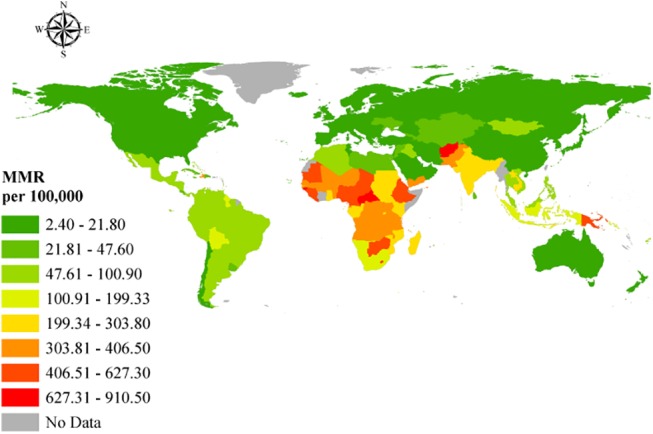
Geographic representation of the MMR. Open source polygon feature data for the maps were downloaded [20] and, subsequently, projected using ESRI ArcGIS [21].

**Fig 4:**
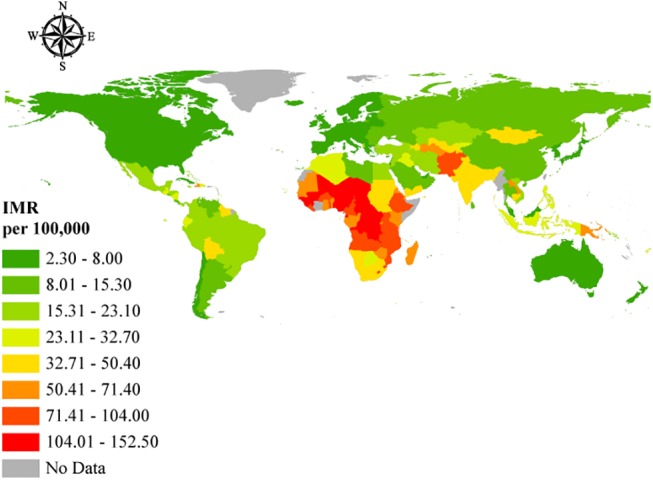
Geographic representation of the IMR. Open source polygon feature data for the maps were downloaded [20] and, subsequently, projected using ESRI ArcGIS [21].

**Fig 5:**
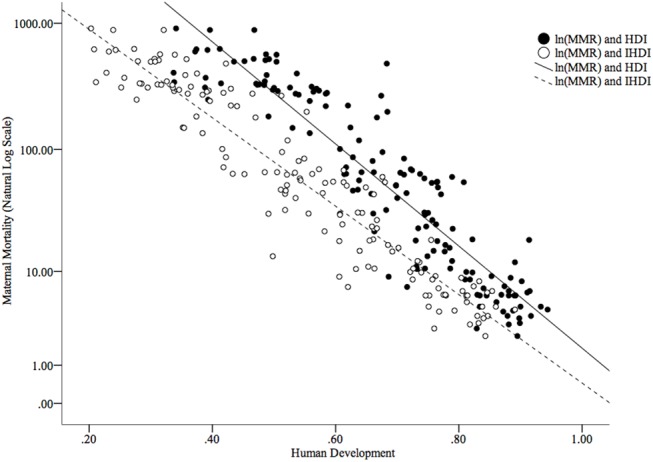
Scatterplot of MMR (on a natural logarithmic scale) and human development as measured by the HDI and IHDI.

**Fig 6:**
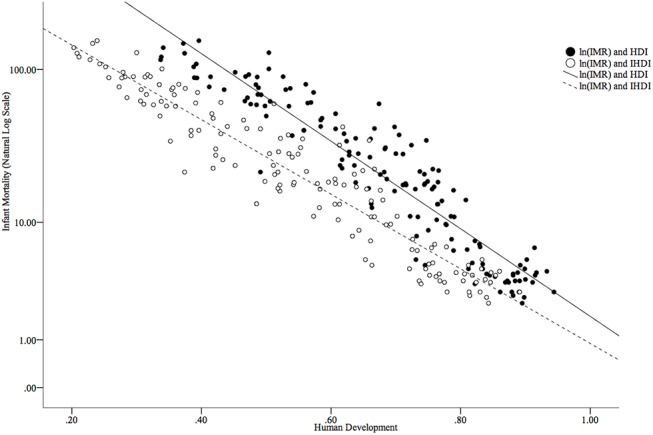
Scatterplot of IMR (on a natural logarithmic scale) and human development as measured by the HDI and IHDI.

The worldwide MMR in 2013 was 209.1 per 100,000 live births; however, this rate was not equally distributed around the world (Fig 3). The global IMR (under 5 years) in 2013 was 44.0 per 1,000 live births from 2.4 per 1,000 live births in Iceland to 152.5 per 1,000 live births in Guinea-Bissau (Fig 4). Figs 5 and 6 show how the IMR and MMR decrease as human development increases.

Regarding the infant mortality gradation variables (i.e., ENMR, LNMR, and PNMR), we also explored ranges. Singapore had the lowest ENMR with 0.8, while Mali had the highest ENMR with 31.4. The LNMR was lowest in Sweden (LNMR = 0.3) and highest in Guinea-Bissau (LNMR = 12.3). Lastly, an analysis of the minimum and maximum values of the PNMR by country revealed that, much like the ENMR, Singapore had the lowest PNMR (PNMR = 0.8) and the Central African Republic had the highest PNMR (PNMR = 50.3).

### Analytical Data

Maternal and infant mortality were both strongly and negatively correlated with both HDI and IHDI (Table 1); however, Steiger’s Z test–which transforms correlation coefficients into *Z* scores and subsequently, with Steiger’s [19] equations numbered three and ten, permits the computation of the asymptotic covariance of the estimates–for the equality of two dependent correlations revealed that IHDI was more strongly correlated than HDI with MMR (*Z* = 4.897, *p* < 0.001), IMR (*Z* = 2.524, *p* = 0.012), ENMR (*Z* = 2.936, *p* = 0.003), LNMR (*Z* = 2.272, *p* = 0.023), and PNMR (*Z* = 2.277, *p* = 0.023).

**Table 1:** Bivariate Pearson Correlation Coefficients (N = 145).

	1	2	3	4	5	6
1. HDI						
2. IHDI	.978					
	[.969, .985]					
3. MMR	-.919	-.947				
	[-.940, -.896]	[-.960, -.932]				
4. IMR	-.935	-.949	.942			
	[-.951, -.919]	[-.962, -.935]	[.922, .964]			
5. ENMR	-.894	-.915	.925	.978		
	[-.917, -.870]	[-.937, -.892]	[.898, .950]	[.968, .985]		
6. LNMR	-.901	-.917	.909	.963	.942	
	[-.930, -.866]	[-.946, -.878]	[.848, .949]	[.914, .984]	[.897, .970]	
7. PNMR	-.923	-.937	.923	.989	.950	.951
	[-.943, -.902]	[-.953, -.919]	[.892, .952]	[.983, .993]	[.925, .968]	[.901, .977]

*Note*. Bootstrap results were based on 1,000 bootstrap samples. Bias corrected 95% confidence intervals are displayed in parentheses. All correlations were based on natural logarithmic transformations of the MMR, IMR, ENMR, LNMR, and PNMR variables.

Simple OLS regression models showed that IHDI was a better predictor of infant and maternal mortality than HDI. In Table 2, side-by-side regression models are shown for MMR, IMR, ENMR, LNMR, and PNMR with HDI and IHDI as predictor variables. In each case, the variance explained in the dependent variable by the predictor variable was greater for the IHDI. Specifically, when IHDI was used as the predictor variable instead of HDI, the *R* ^*2*^ value was 0.053 higher for MMR, 0.025 higher for IMR, 0.038 higher for ENMR, 0.029 higher for LNMR, and 0.026 higher for PNMR.

**Table 2:** Simple OLS Regression Models for MMR, IMR, ENMR, LNMR, and PNMR on HDI and IHDI.

					BCa 95% CI					
		B	Bias	SE(B)	Lower	Upper	*F*	*df*	*p*	*R* ^*2*^
MMR										
1	HDI	-9.618	-.001	.330	-10.316	-8.972	773.046	(1, 143)	< .001	.844
	Intercept	10.456	.001	.235	10.023	10.937				
2	IHDI	-8.344	-.002	.193	-8.730	-7.987	1,238.889	(1, 143)	< .001	.897
	Intercept	8.519	.004	.115	8.309	8.750				
IMR										
1	HDI	-6.801	-.001	.192	-7.207	-6.416	999.453	(1, 143)	< .001	.875
	Intercept	7.605	.003	.133	7.367	7.882				
2	IHDI	-5.807	.005	.123	-6.050	-5.540	1,289.726	(1, 143)	< .001	.900
	Intercept	6.183	.000	.068	6.051	6.323				
ENMR										
1	HDI	-5.715	-.012	.240	-6.187	-5.312	571.723	(1, 143)	< .001	.800
	Intercept	5.802	.008	.162	5.524	6.151				
2	IHDI	-4.923	-.007	.149	-5.228	-4.633	740.319	(1, 143)	< .001	.838
	Intercept	4.632	.003	.079	4.488	4.800				
LNMR										
1	HDI	-5.381	.005	.233	-5.808	-4.890	618.882	(1, 143)	< .001	.812
	Intercept	4.477	-.002	.152	4.159	4.770				
2	IHDI	-4.607	.000	.175	-4.929	-4.237	753.951	(1, 143)	< .001	.841
	Intercept	3.359	.002	.089	3.179	3.537				
PNMR										
1	HDI	-7.208	-.012	.221	-7.655	-6.785	819.267	(1, 143)	< .001	.851
	Intercept	6.660	.011	.158	6.358	6.992				
2	IHDI	-6.158	.007	.145	-6.438	-5.854	1,019.992	(1, 143)	< .001	.877
	Intercept	5.155	-.003	.083	4.993	5.307				

*Note*. Bootstrap results are based on 1,000 bootstrap samples. All regression models are based on logarithmic transformations of the MMR, IMR, ENMR, LNMR, and PNMR variables.

## Discussion

Health disparities are preventable differences in the burden of disease, injury, and violence, and represent opportunities to achieve optimal health for socially disadvantaged populations [22]. They lead to conspicuous differences in health outcomes across sub-groups within the same population and are often linked to social, economic, or environmental disadvantages [23]. Inequalities in the distribution and access to socio-economic, healthcare, and environmental resources, with attendant influences on health and health-seeking behavior contribute immensely to the stratification of morbidity and mortality statistics within, without, and across populations the world over [24]. While composite indices–such as the HDI–offer some predictive value on the importance of human development in terms of the health of individuals and populations, the use of national aggregates and averages in the computation of such indices might conceal important disparities in populations that might hold some clues to improving health and health outcomes among socially and economically disadvantaged individuals and sub-groups within the larger populations.

HDI for the countries included in the study depicted a significant range. When adjusted for inequalities, there were changes in the positions that some of the countries occupied in the HDI ranking–as mentioned in the results section. The considerable percentage differences between HDI and IHDI for some of the countries further supports the weight of inequalities in the factors determining human development in the affected countries. For instance, a relatively high GDP in a country does not mean that an even distribution of income exists within that country. Indeed, a few people with excess wealth may push up the GDP, obliterating the true picture of the many people living below the poverty line, and when factored into the HDI, will give the impression the country is doing very well. When the inequalities in these factors are examined, loss to human development in these countries is realized, giving a better, more realistic view of the situation on the ground. The percentage differences in HDI and IHDI were even more dramatic among the bottom 43 countries, with some experiencing values as high as 44.3 percent. By looking at Figs 5 and 6, it is clear to see that both higher HDI and IHDI are negatively correlated with maternal and childhood mortality. Indeed, all sub-classes of IMR as well as maternal mortality were higher among countries with lower HDI and IHDI. Considering the trend, as observed in the results, where countries moved down the ladder after adjusting for inequalities–having lost appreciable percentage points and in default, were more likely to have more maternal and childhood deaths for all sub-groups–it might be sufficient to connect higher IMR and MMR with widening inequalities across countries. From the results and ensuing analyses, the inequalities could play a critical role in the distribution of maternal and childhood deaths across countries, especially in the countries with lower human development.

By examining Figs 3 and 4, it can be readily observed that the highest MMRs and IMRs were seen on the African continent, with the least seen in Western Europe and North America. This correlates well with the low HDI rankings associated with the African countries in the study. Given that relatively higher percentage differences exist between HDI and IHDI in these bottom rankings, where the majority of African countries lie per the study findings, suggests wider disparities in the distribution of socio-economic, healthcare, and environmental variables that directly and indirectly affect MMR and IMR. Gaping inequalities in income, education, healthcare, human and environmental resources on the African continent has led to poor maternal and childhood outcomes among poor, rural individuals and communities. The much publicized 4 delays (4Ds)—delays in recognizing danger signs of labor due to reduced availability of skilled labor; delays in the decision to seek help because of limited knowledge and capacity to do so; delays in getting to the health facility due to poor road networks and poor ambulance and transportation services; and delays within the health facilities mainly due to lack of emergency preparedness, logistical and human resource challenges have culminated in the higher MMRs recorded in much of Sub-Saharan Africa [25]. The situation for childhood mortality is no different, where lack of formal education among poor, rural dwellers, limited healthcare, and human resources and diminished incomes coupled with poor health-seeking behaviors and socio-cultural practices have contributed significantly to high childhood mortality rates in these areas. Not surprisingly, despite some gains in the achievement of Millenium Development Goals (MDGs) 4 and 5, many such countries in Africa and elsewhere, where there are vast inequalities in these social indicators, have failed to meet their targets and may likely continue to miss them until deliberate policies are implemented to address these disparities. Indeed the United Nations conceded in its 2015 report on the state of the Millennium Development Goals that “despite many successes, the poorest and most vulnerable people are being left behind” [26]. In countries where these inequalities are relatively limited, the IHDI is better, with attendant low levels of MMRs and IMRs, suggesting in many respects, good access to and affordability of healthcare resources and health information utilization owing to better education, resulting in better health-seeking behavior that together reduce MMR and IMR.

This paper has various strengths, such as the use of ArcGIS mapping to highlight locations with high MMR and IMR and associated HDIs and IHDIs, and the determination of how inequalities affect the ranking of countries by human development and how said development influences the distribution of maternal and childhood mortality. Nonetheless, some weaknesses have been observed in this study. We also considered the challenges in getting the data into a normal distribution as a limitation of some degree; however, given that the data was transformed and bootstrapped, some of these challenges were alleviated. Limitations on the actual distribution of maternal and childhood deaths in the countries by location and related factors may have also been a weakness in the analysis.

## Conclusions

In the final analysis, the results of the present study supported our hypothesis: that even when both the HDI and the IHDI correlate with the infant and maternal mortality rates, the IHDI is a better predictor for these two health indicators. Pregnant women and children continue to be priority populations for many countries, and while direct intervention programs to address morbidity and mortality issues among these groups are prudent, social programs that address inequalities in education, income, access to healthcare resources and related factors are imperative and of equal importance in reducing mortality among these populations in the medium to long term especially, but also in the short term.

## Supporting Information

S1 Table Data for IHDI, HDI, MMR, IMR, ENMR, LNMR, PNMR. (XLS)
